# Supplementary material for: Mechanical force regulates ligand binding and function of PD-1
Source: Nat Commun. 2024 Sep 27;15:8339. doi: 10.1038/s41467-024-52565-2 (PMC11437077; doi:10.1038/s41467-024-52565-2)
Supplement: Supplementary file 1 — Supplementary Information [file 41467_2024_52565_MOESM1_ESM.pdf]

# Supplementary Figures for

## Mechanical force regulates ligand binding and function of PD-1

Kaitao Li[1,2†], Paul Cardenas-Lizana[1,2‡], Jintian Lyu[1,2‖], Anna V. Kellner[1&], Menglan Li[1,2], Peiwen Cong[1,2], Valencia E. Watson[1,2], Zhou Yuan[1-3§], Eunseon Ahn[4,5¶], Larissa Doudy[1,2], Zhenhai Li[1,3#], Khalid Salaita[1,6], Rafi Ahmed[4,5], Cheng Zhu[1-3]*

[1]Wallace H. Coulter Department of Biomedical Engineering, [2]Parker H. Petit Institute for Bioengineering and Biosciences, [3]George W. Woodruff School of Mechanical Engineering, Georgia Institute of Technology, Atlanta, Georgia 30332, USA

[4]Emory Vaccine Center, [5]Department of Microbiology and Immunology, Emory University School of Medicine, [6]Department of Chemistry, Emory University, Atlanta, GA 30322, USA

*Corresponding author, **Email:** cheng.zhu@bme.gatech.edu

[†]Present address: Shennon Biotechnologies, San Francisco, CA, USA

[‡]Present address: Department of Bioengineering and Chemical Engineering, University of Engineering and Technology - UTEC, Lima, Peru

[‖]Present address: L.E.K. consulting, Boston, MA, USA

[&]Present address: Elephas, Madison, WI, USA

[§]Present address: Alfred E. Mann Department of Biomedical Engineering, University of Southern California, Los Angeles, CA, USA

[¶]Present address: Merck, South San Francisco, CA, USA

[#]Present address: School of Mechanics and Engineering Science, Shanghai University, Shanghai, China

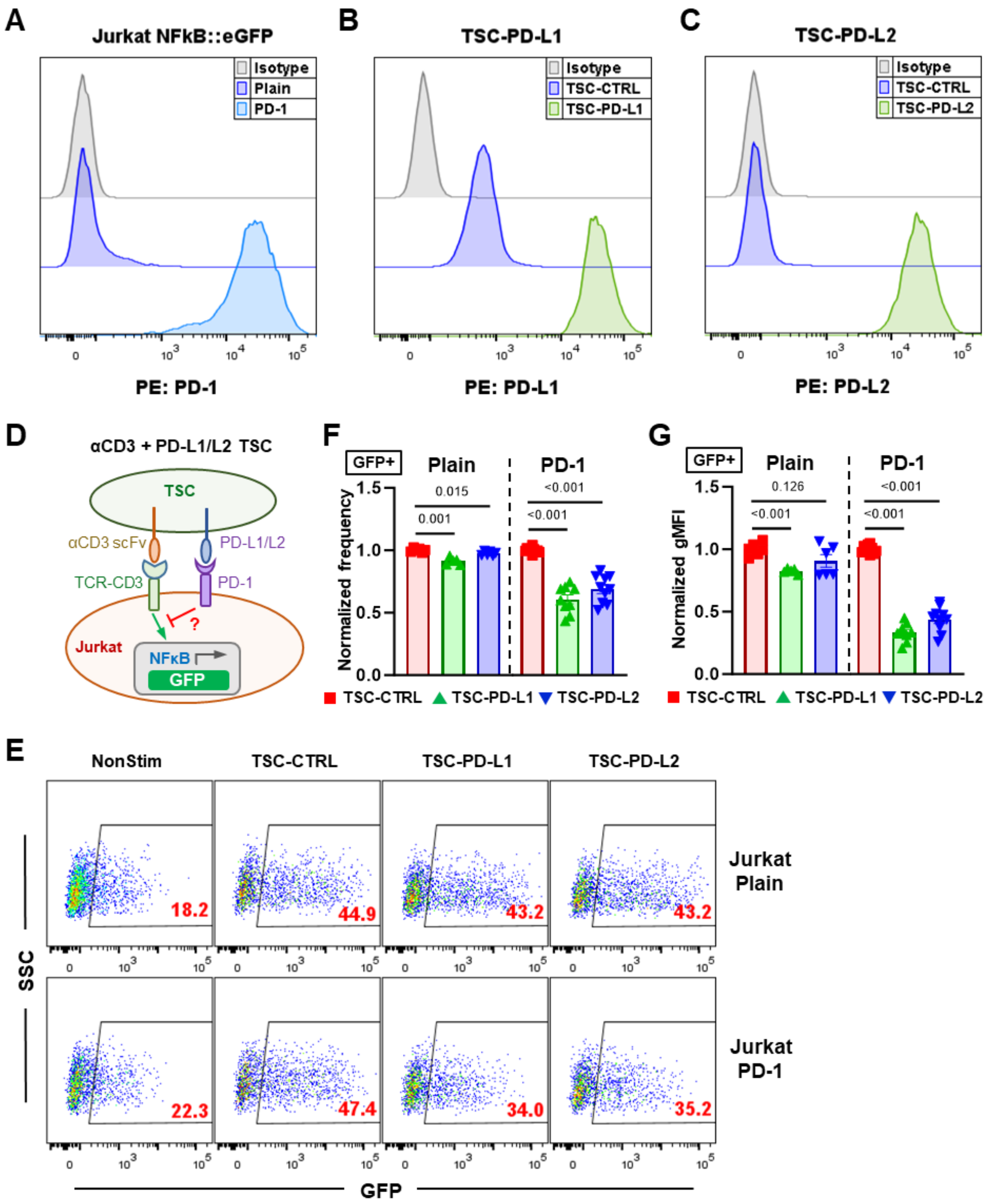


**Fig. S1.** Detection of PD-1 mediated suppression of TCR-CD3 signaling using NFκB::eGFP reporter Jurkat cells. (A) Flow cytometry histograms of PD-1 staining of plain or PD-1 transduced NFκB::eGFP reporter Jurkat cells. (B-C) Flow cytometry histograms of PD-L1 (B) or PD-L2 (C) staining of TSC without transducing PD-1 ligands (TSC-CTRL) or transduced with PD-L1 (TSC-

PD-L1) or PD-L2 (TSC-PD-L2). (D) Schematics of stimulating NFκB::eGFP reporter Jurkat cells with T-cell stimulator cells (TSC) expressing a single-chain fragment variable (scFv) of anti-CD3 (clone OKT3) and PD-L1 or PD-L2. (E) Representative SSC *vs* GFP plots of reporter Jurkat cells 24 hr after stimulation with indicated conditions. (F-G) Quantification of GFP expression for conditions in B. n = 5 - 6 for plain pooled from 3 independent experiments or n = 9 - 10 for PD-1 reporter cells pooled from 5 independent experiments. Normalized frequency (F) and normalized geometric mean fluorescence intensity (gMFI) (G) were calculated as (sample – averaged background)/(TSC control – averaged background) and presented as mean +/- SEM. Numbers on graphs represent p values calculated from two-tailed student t-test. Source data are provided in Source Data file.

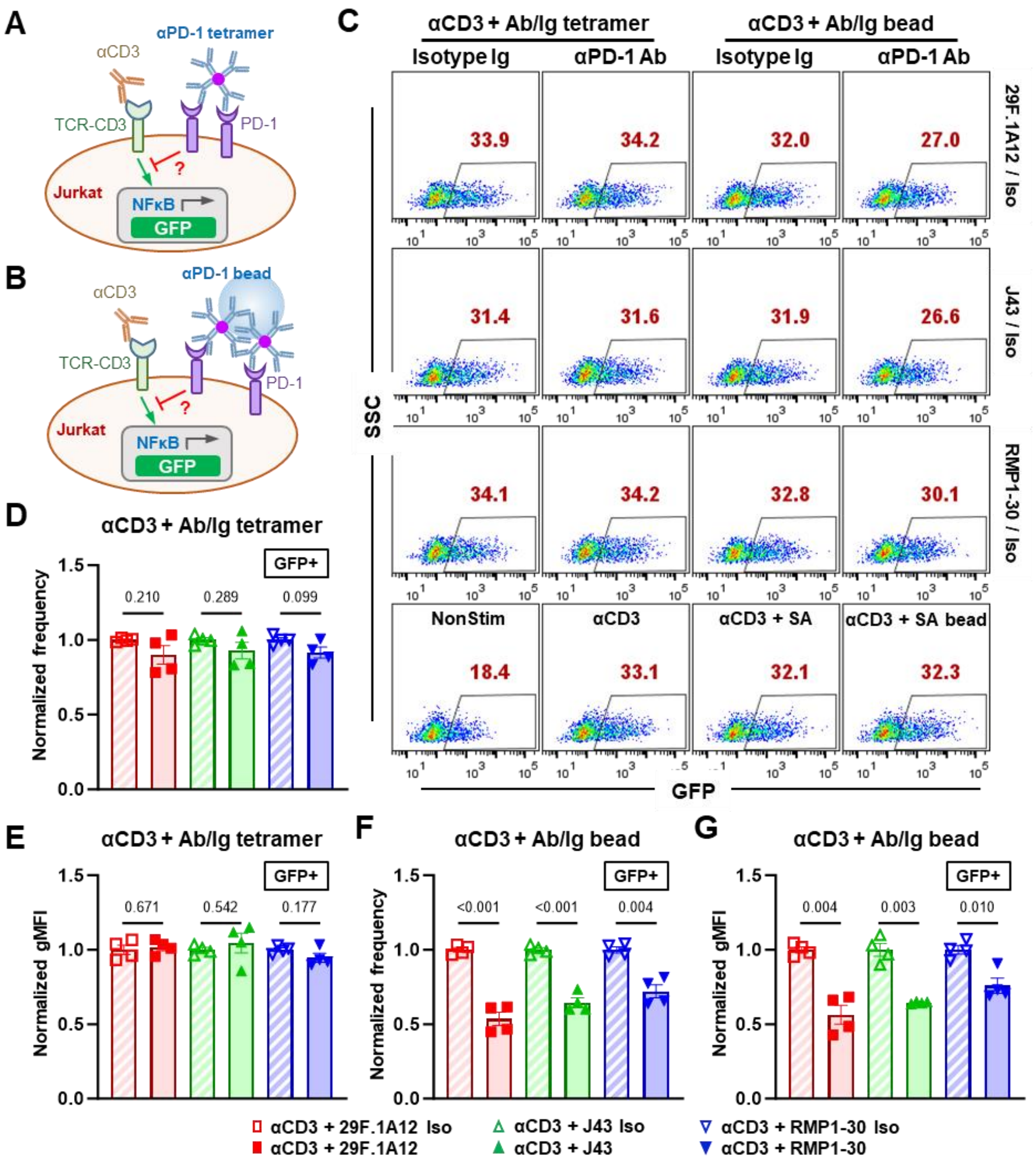


**Fig. S2.** Immobilized anti-PD-1 antibody triggers PD-1 function. (A&B) Schematics of stimulating NFκB::eGFP reporter Jurkat cells with soluble anti-CD3 and soluble (A) or bead-coated (B) anti-PD-1 antibody (or isotype Ig). (C) Representative SSC *vs* GFP plots of reporter Jurkat cells 24 hr after stimulation with indicated conditions. (D-G) Quantification of GFP expression for conditions in A. n = 4 for all conditions pooled from two independent experiments. Normalized frequency (D&F) and normalized gMFI (E&G) were calculated as (sample – averaged background)/(isotype control

– averaged background) and presented as mean +/- SEM. Numbers on graphs represent p values calculated from two-tailed student t-test. Source data are provided in Source Data file.

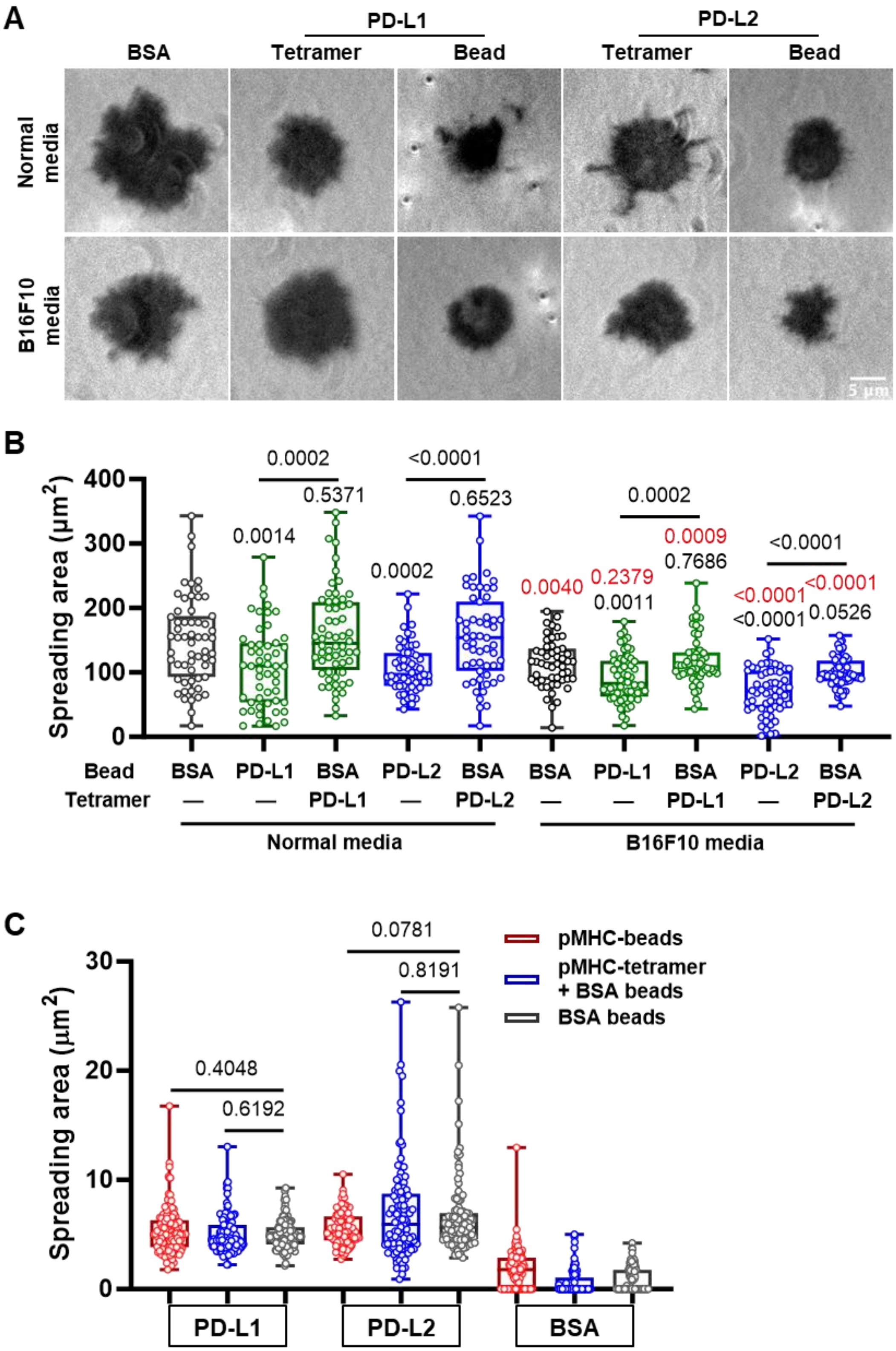
A
PD-L1
PD-L2
BSA
Tetramer
Bead
Tetramer
Bead
Normal media
B16F10 media
5 µm
B
Spreading area (µm2)
0.0002
<0.0001
0.5371
0.6523
0.0014
0.0002
0.0002
0.0009
0.7686
<0.0001
0.0040
0.2379
0.0011
<0.0001
<0.0001
<0.0001
0.0526
Bead
Tetramer
BSA
—
PD-L1
—
BSA
PD-L1
PD-L2
—
BSA
PD-L2
BSA
—
PD-L1
—
BSA
PD-L1
PD-L2
—
BSA
PD-L2
Normal media
B16F10 media
C
Spreading area (µm2)
0.0781
0.8191
0.4048
0.6192
pMHC-beads
pMHC-tetramer + BSA beads
BSA beads
PD-L1
PD-L2
BSA

**Fig. S3.** Respective effects of tumor cell media and TCR stimulation on T cell spreading on pMHC and on PD-Ligands, respectively. Representative RICM images (A) and quantification (B) of activated $CD8^+$ OT1 T cells spreading on SIINFEKL:H2-$K^b$ coated surface in contact with beads coated with PD-L1, PD-L2, or BSA in the presence of tetrameric PD-L1, PD-L2, or BSA in solution. The experiment was done in either normal media (*upper row*, pH = 7.76) or B16F10 melanoma cell-conditioned media (*lower row*, pH = 7.02). n = 59, 52, 60, 57, 55, 51, 60, 57, 60, and 59 cells. Black numbers on graphs represent p values calculated from two-tailed Mann-Whitney U test of indicated two groups or PD-Ligand groups (green or blue) with BSA control (black). Red numbers on graphs represent p values calculated from two-tailed Mann-Whitney U test of normal media vs B16F10 media for each group. (C) Engagement of TCR with bead-bound or tetrameric pMHC in solution had no effect on T cell spreading on PD-L1/L2-coated surface. Activated OT1 T cells spreading on PD-L1, PD-L2, or BSA surface in contact with SIINFEKL:H2-$K^b$ or BSA coated beads in the absence of presence of tetrameric SIINFEKL:H2-$K^b$ in solution. n = 119, 115, 121, 109, 105, 111, 110, 102, and 106 cells from 1 of 2 independent experiments. Data were presented in box (median with 25%/75% boundaries) and whisker (min and max) plots. Numbers on graphs represent p values calculated from two-tailed Mann-Whitney U test of indicated two groups or the experiment group (green or blue) with BSA control (black). Source data are provided in Source Data file.

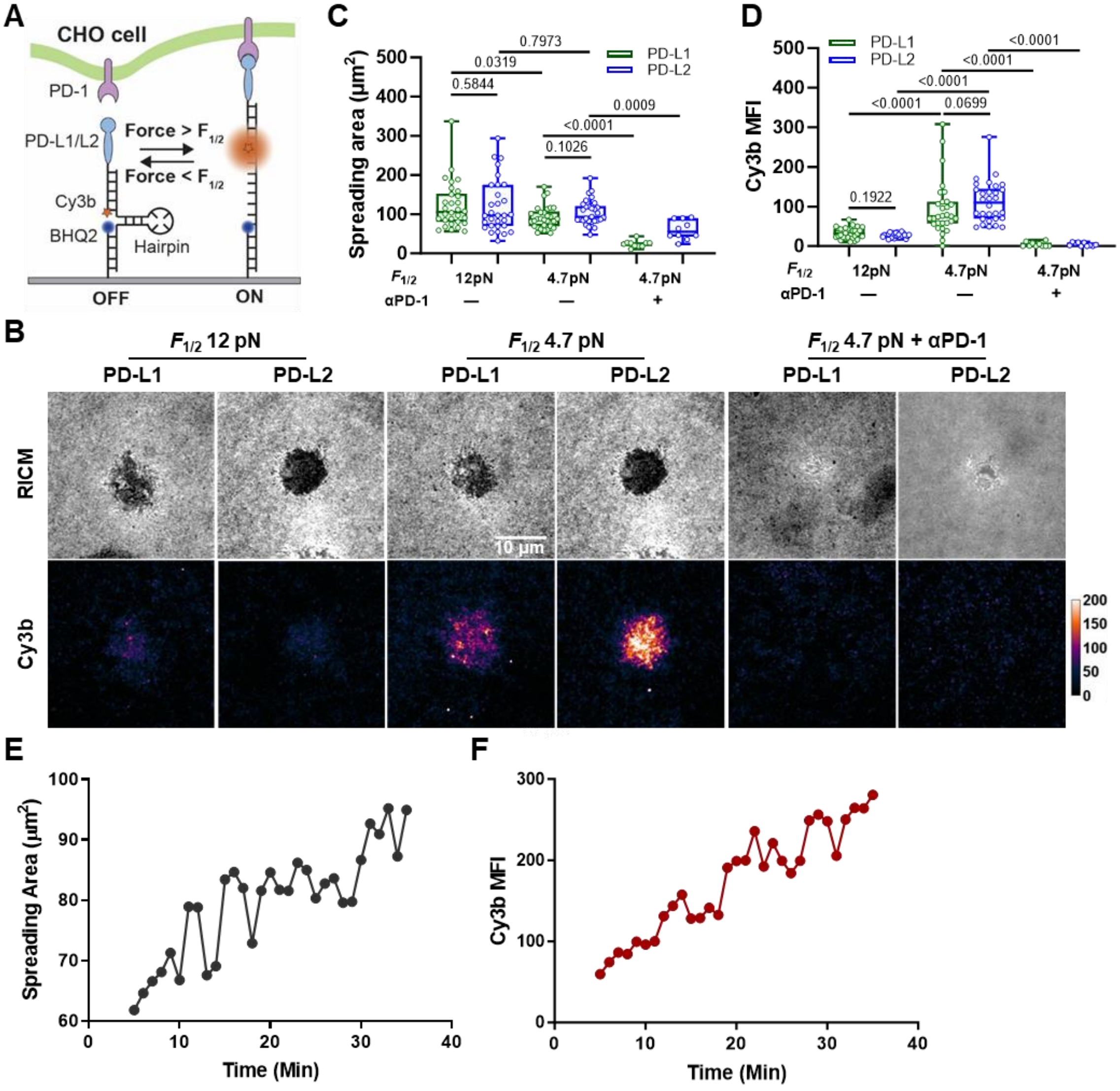


**Fig. S4.** DNA-based MTPs reveal active cellular forces applied to PD-1–PD-Ligand bonds. (A) Schematics of detecting cellular forces on PD-1–PD-Ligand bonds using DNA-based molecular tension probes (MTPs). Forces above threshold unfold the hairpin, which separates Cy3B from the BHQ2 to de-quench the fluorophore. (B) Representative reflection interference contrast microscopy (RICM) and total internal reflection fluorescence (TIRF) images of PD-1 expressing CHO cells 30 min after landing on glass surface functionalized with MTPs of indicated conditions. For PD-1 blockade, cells were pre-incubated with PD-1 blocking antibody clone 29F.1A12 before imaging. (C-D) Quantification of cell spreading area (C) and tension signal (D) for conditions in B. n = 30,

30, 30, 30, 10, and 10 pooled from 3 independent experiments. Data were presented in box (median with 25%/75% boundaries) and whisker (min and max) plots. Numbers on graphs represent p values calculated from two-tailed Mann-Whitney U test. (E&F) Realtime quantification of spreading area (E) and Cy3b fluorescence (F) of a PD-1 CHO cell spreading on PD-L2-coupled MTP of 4.7 pN threshold force as shown in Video S2. Source data are provided in Source Data file.

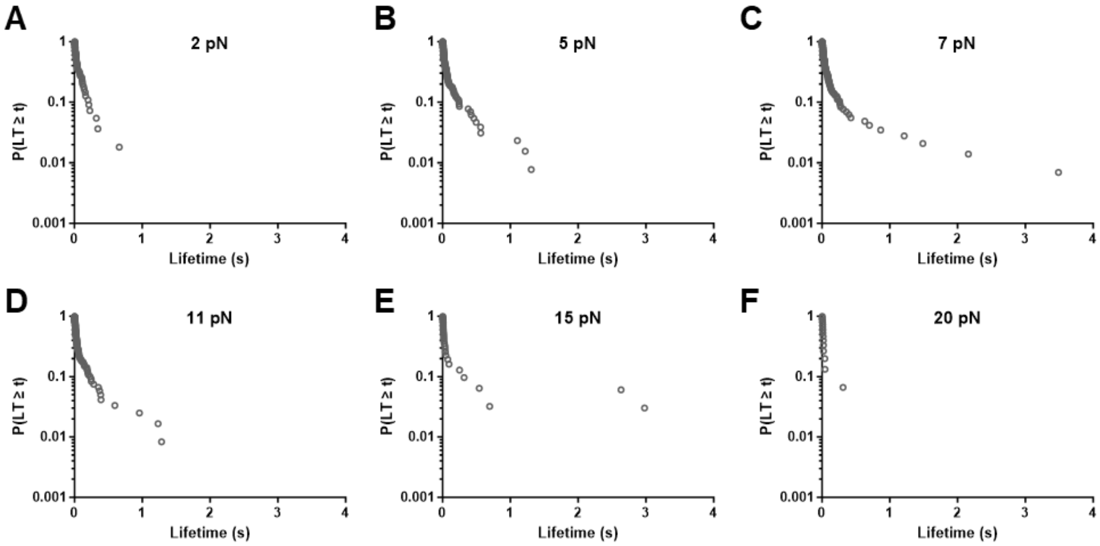


**Fig. S5.** Survival frequencies (fraction of event with lifetime greater than *t*) of PD-1–PD-L1 bond lifetime for different force bins. n = 55, 129, 144, 120, 33, and 16 lifetime events for (A) to (F) respectively. Source data are provided in Source Data file.

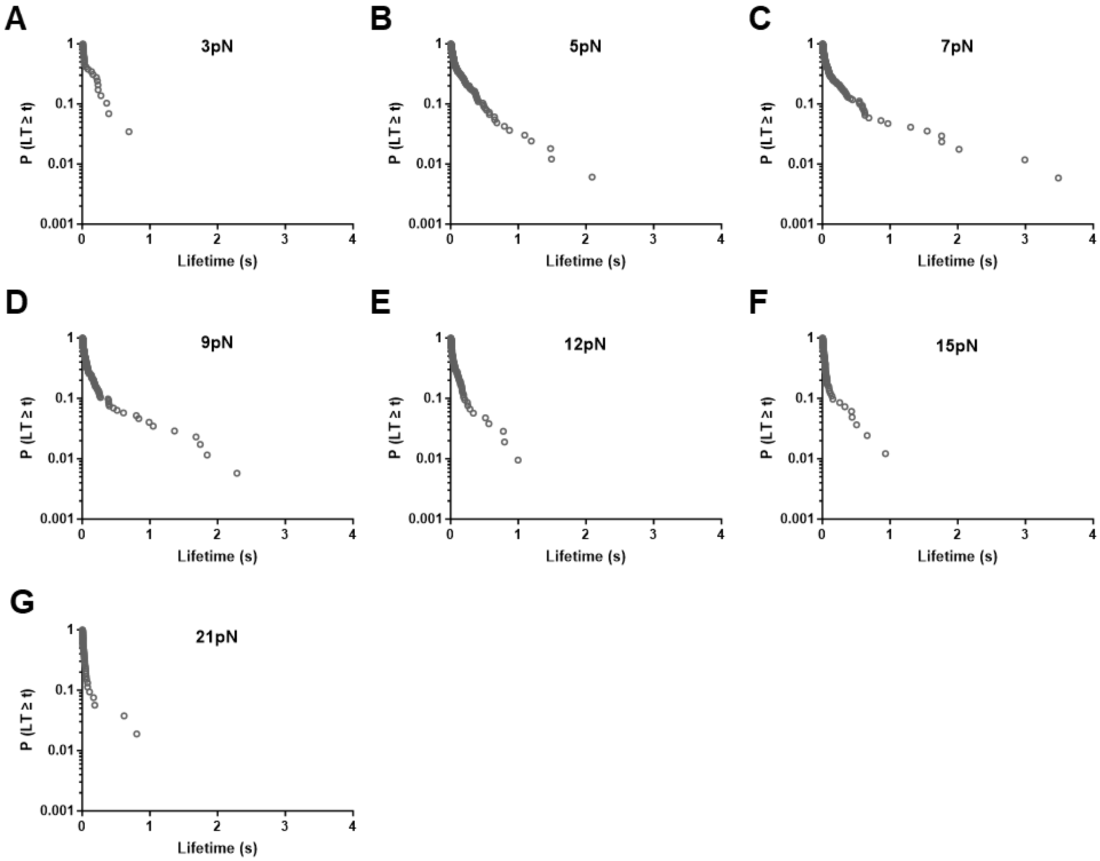


**Fig. S6.** Survival frequencies (fraction of event with lifetime greater than *t*) of PD-1–PD-L2 bond lifetime for different force bins. n = 29, 165, 170, 173, 105, 82, and 53 lifetime events for (A) to (G) respectively. Source data are provided in Source Data file.

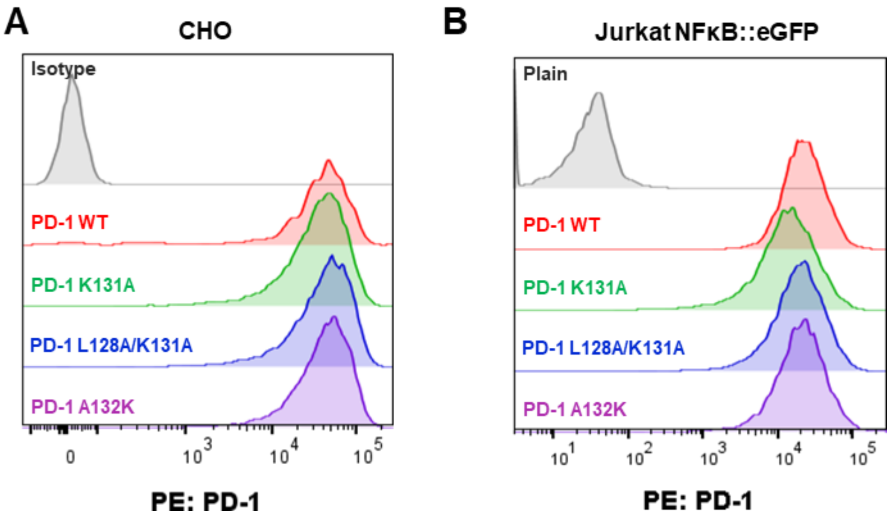


**Fig. S7.** Flow cytometry histograms comparing PD-1 staining of CHO cells (A) or NFκB::eGFP reporter Jurkat cells (B) expressing WT or indicated mutants of PD-1.

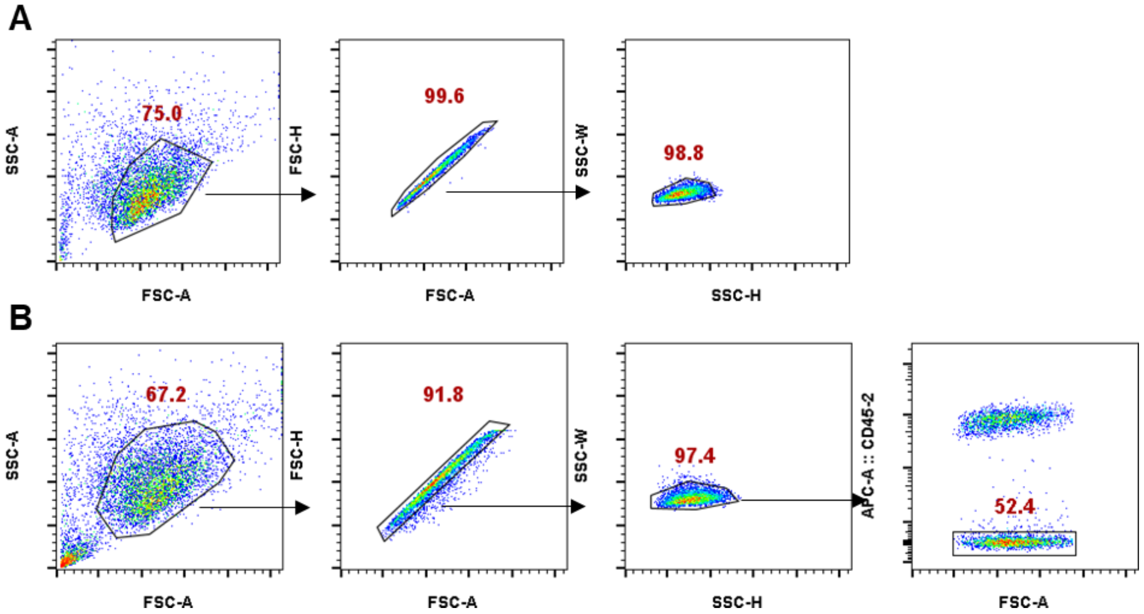


**Fig. S8.** Gating strategies for flow cytometry data. (A) Sequential gating for data with one cell population to quantify GFP induction, or expression of surface PD-1, PD-L1, or PD-L2 (related to Figs. 1, S1A-C, S2, and S7). (B) Sequential gating or reporter Jurkat cells co-cultured with CD45.2+ TSC cells (related to Figs. 7, and S1E-G).
