# Supplementary material for: Mechanical force regulates ligand binding and function of PD-1
Source: Nat Commun. 2024 Sep 27;15:8339. doi: 10.1038/s41467-024-52565-2 (PMC11437077; doi:10.1038/s41467-024-52565-2)
Supplement: Supplementary file 5 — Reporting Summary [file 41467_2024_52565_MOESM5_ESM.pdf]

nature portfolio

Corresponding author(s): Cheng Zhu

Last updated by author(s): Sep 2, 2024

Reporting Summary

Nature Portfolio wishes to improve the reproducibility of the work that we publish. This form provides structure for consistency and transparency in reporting. For further information on Nature Portfolio policies, see our Editorial Policies and the Editorial Policy Checklist.

Statistics

For all statistical analyses, confirm that the following items are present in the figure legend, table legend, main text, or Methods section.

n/a	Confirmed	
☐	☒	The exact sample size (*n*) for each experimental group/condition, given as a discrete number and unit of measurement
☐	☒	A statement on whether measurements were taken from distinct samples or whether the same sample was measured repeatedly
☐	☒	The statistical test(s) used AND whether they are one- or two-sided<br>*Only common tests should be described solely by name; describe more complex techniques in the Methods section.*
☐	☒	A description of all covariates tested
☐	☒	A description of any assumptions or corrections, such as tests of normality and adjustment for multiple comparisons
☐	☒	A full description of the statistical parameters including central tendency (e.g. means) or other basic estimates (e.g. regression coefficient) AND variation (e.g. standard deviation) or associated estimates of uncertainty (e.g. confidence intervals)
☐	☒	For null hypothesis testing, the test statistic (e.g. *F*, *t*, *r*) with confidence intervals, effect sizes, degrees of freedom and *P* value noted<br>*Give P values as exact values whenever suitable.*
☒	☐	For Bayesian analysis, information on the choice of priors and Markov chain Monte Carlo settings
☒	☐	For hierarchical and complex designs, identification of the appropriate level for tests and full reporting of outcomes
☒	☐	Estimates of effect sizes (e.g. Cohen's *d*, Pearson's *r*), indicating how they were calculated

Our web collection on statistics for biologists contains articles on many of the points above.

Software and code

Policy information about availability of computer code

Data collection	Labview 2016, FACSDiva v9
Data analysis	Labview 2016, Labview 2019, Matlab 2020a, Microsoft Excel 2016, Flowjo v10, GraphPad Prism 10, VMD v1.9.3

For manuscripts utilizing custom algorithms or software that are central to the research but not yet described in published literature, software must be made available to editors and reviewers. We strongly encourage code deposition in a community repository (e.g. GitHub). See the Nature Portfolio guidelines for submitting code & software for further information.

Data

Policy information about availability of data

All manuscripts must include a data availability statement. This statement should provide the following information, where applicable:

- Accession codes, unique identifiers, or web links for publicly available datasets
- A description of any restrictions on data availability
- For clinical datasets or third party data, please ensure that the statement adheres to our policy

Data supporting the findings of this study are presented in the article and supplementary materials and are available from the corresponding authors upon request. Source data are provided with this paper.

Research involving human participants, their data, or biological material

Policy information about studies with human participants or human data. See also policy information about sex, gender (identity/presentation), and sexual orientation and race, ethnicity and racism.

Reporting on sex and gender	N/A
Reporting on race, ethnicity, or other socially relevant groupings	N/A
Population characteristics	N/A
Recruitment	N/A
Ethics oversight	N/A

Note that full information on the approval of the study protocol must also be provided in the manuscript.

Field-specific reporting

Please select the one below that is the best fit for your research. If you are not sure, read the appropriate sections before making your selection.

[x] Life sciences [ ] Behavioural & social sciences [ ] Ecological, evolutionary & environmental sciences

For a reference copy of the document with all sections, see nature.com/documents/nr-reporting-summary-flat.pdf

Life sciences study design

All studies must disclose on these points even when the disclosure is negative.

Sample size	No sample size calculation was used to predetermine the sample size to be tested. However, expected sample sizes were understood from observing the variance within experiments as well as from leveraging previous publications as commonly accepted in the filed of study.
Data exclusions	No data were excluded from the analysis.
Replication	Replications were performed 2-5 times as indicated in figure legend. All attempts of replication were successful.
Randomization	For all experiments cells of the same condition were randomized into control and different experimental groups for treatment and analysis.
Blinding	Blinding was not applied due to the complexity of sample preparation and experiment procedure. However, all procedures were applied equally to all samples. We also we had independent person other than the experimenter to analyze the data.

Reporting for specific materials, systems and methods

We require information from authors about some types of materials, experimental systems and methods used in many studies. Here, indicate whether each material, system or method listed is relevant to your study. If you are not sure if a list item applies to your research, read the appropriate section before selecting a response.

Materials & experimental systems

n/a	Involved in the study	
[ ]	[x]	Antibodies
[ ]	[x]	Eukaryotic cell lines
[x]	[ ]	Palaeontology and archaeology
[ ]	[x]	Animals and other organisms
[x]	[ ]	Clinical data
[x]	[ ]	Dual use research of concern
[x]	[ ]	Plants

Methods

n/a	Involved in the study	
[x]	[ ]	ChIP-seq
[ ]	[x]	Flow cytometry
[x]	[ ]	MRI-based neuroimaging

Antibodies

Antibodies used	Biolegend: PE-anti-PD-1 (RMP1-30, 1:20 dilution), PE-Rat IgG2b,κ (RTK4530, 1:20 dilution), APC-anti-CD45.2 (104, 1:100 dilution), anti-CD3 (OKT3, 10ug/ml) and anti-mouse IgG2a (RMG2a-62, 5ug/ml), biotinylated antiPD-1 (clone 29F.1A12, 15ug/ml), biotinylated antiPD-1 (clone 29F.1A12 and clone RMP1-30, 15ug/ml), and biotinylated isotype controls (Rat IgG2a,κ clone RTK2758 and Rat IgG2b,κ clone RTK4530, 15ug/ml). BD Biosciences: PE-anti-PD-L1 (MIH1, 1:20), PE-Rat IgG2a,λ (557076, 1:20), PE-anti-PD-L2 (TY25, 1:20), PE-Rat IgG2a,κ (R35-95, 1:20). Invitrogen: biotinylated antiPD-1 (J43, 15ug/ml) and isotype control (eBio199Arm, 15ug/ml).
Validation	Anti-PD-1 (clone RMP1-30): IL-4-producing CD4+ T cells in reactive lymph nodes during helminth infection are T follicular helper cells. J Exp Med. 2009 May 11;206(5):1001-7.<br>AntiPD-1 (clone 29F.1A12): PD-1 regulates germinal center B cell survival and the formation and affinity of long-lived plasma cells. Nat Immunol. 2010 Jun;11(6):535-42.<br>AntiPD-1 (clone J43): Agata Y, Kawasaki A, Nishimura H, et al. Expression of the PD-1 antigen on the surface of stimulated mouse T and B lymphocytes. Int Immunol. 1996 May; 8(5):765-772.<br>anti-PD-L1 (clone MIH1): Lenalidomide and programmed death-1 blockade synergistically enhances the effects of dendritic cell vaccination in a model of murine myeloma. Frontiers in immunology 9 (2018): 1370.<br>Anti-PD-L2 (clone TY25): T follicular helper cells contribute to pathophysiology in a model of neuromyelitis optica spectrum disorders. JCI Insight. 2023 Feb 22;8(4):e161003.<br>Anti-CD45.2 (clone 104): Pyrimidine de novo synthesis inhibition selectively blocks effector but not memory T cell development. Nat Immunol. 2023 Mar;24(3):501-515.<br>Anti-CD3 (clone OKT3): Targeting histone methylation to reprogram the transcriptional state that drives survival of drug-tolerant myeloid leukemia persisters. iScience. 2022 Aug 25;25(9):105013.

Eukaryotic cell lines

Policy information about cell lines and Sex and Gender in Research

Cell line source(s)	293T (cat. CRL-3216) and CHO-K1 (cat. CCL-61) cells are from ATCC. Jurat and TSC cells are from Peter Steinberger's lab at Medical University of Vienna.
Authentication	Authentication was not performed.
Mycoplasma contamination	Mycoplasma contamination was not tested.
Commonly misidentified lines<br>(See ICLAC register)	No commonly misidentified cell lines were used in the study.

Animals and other research organisms

Policy information about studies involving animals; ARRIVE guidelines recommended for reporting animal research, and Sex and Gender in Research

Laboratory animals	14-16-week-old OT1 TCR transgenic mice (C57BL/6-Tg(TcraTcrb)1100Mjb/J) obtained from Charles River Laboratory (Lyon, France) and bred in house at the Georgia Institute of Technology.
Wild animals	N/A
Reporting on sex	5 males and 3 females.
Field-collected samples	N/A
Ethics oversight	All experiments in this study were conducted following the protocols approved by the Institutional Review Board (IRB) and Institutional Care and Use Committee (IACUC) of the Georgia Institute of Technology.

Note that full information on the approval of the study protocol must also be provided in the manuscript.

Plants

Seed stocks	N/A
Novel plant genotypes	N/A
Authentication	N/A

Flow Cytometry

Plots

Confirm that:

- [x] The axis labels state the marker and fluorochrome used (e.g. CD4-FITC).
- [x] The axis scales are clearly visible. Include numbers along axes only for bottom left plot of group (a 'group' is an analysis of identical markers).
- [x] All plots are contour plots with outliers or pseudocolor plots.
- [x] A numerical value for number of cells or percentage (with statistics) is provided.

Methodology

Sample preparation	Cells were stained in FACS buffer with corresponding antibodies for 30min at 4 degree, and washed for 3 times with FACS buffer.
Instrument	BD FACS Aria, LSRII, Fortessa
Software	FACSDiva9,Flowjo V10
Cell population abundance	Single population cell lines (100%).
Gating strategy	Cells were gated on FSC vs SSC and then FSC-A vs FSC-H. Subsequent gating was performed based on negative population and controls.

- [x] Tick this box to confirm that a figure exemplifying the gating strategy is provided in the Supplementary Information.
