# Supplementary material for: Mechanical force regulates ligand binding and function of PD-1
Source: Nat Commun. 2024 Sep 27;15:8339. doi: 10.1038/s41467-024-52565-2 (PMC11437077; doi:10.1038/s41467-024-52565-2)
Supplement: Supplementary file 6 — Description of Additional Supplementary Files [file 41467_2024_52565_MOESM6_ESM.pdf]

Title: Supplementary Movie 1.

Description: Realtime Fura-2 340/380 ratiometric pseudo-colar movie of an activated OT1 T cell stimulated with SIINFEKL:H2-K^b-coated RBC and in contact with a BSA coated bead using the fMAF setup.

Title: Supplementary Movie 2.

Description: Realtime RICM and TIRF images of a PD-1 CHO cell spreading on PD-L2-coupled tension probe of 4.7 pN threshold force.

Title: Supplementary Movie 3.

Description: SMD simulated trajectory for pulling the mPD-1–mPD-L2 complex. The C-terminal residue of mPD-1 was harmonically constrained and the C terminus of mPD-L2 was pulled by a dummy spring moving at ∼0.1 nm/ns with a spring constant of ∼70 pN/nm.
